# FDG kinetic modeling in small rodent brain PET: optimization of data acquisition and analysis

**DOI:** 10.1186/2191-219X-3-61

**Published:** 2013-08-06

**Authors:** Malte F Alf, Marianne I Martić-Kehl, Roger Schibli, Stefanie D Krämer

**Affiliations:** 1 Center for Radiopharmaceutical Sciences of ETH, PSI, and USZ, Institute of Pharmaceutical Sciences, Department of Chemistry and Applied Biosciences, ETH Zurich, Zurich CH-8093, Switzerland; 2 Collegium Helveticum ETH and UZH, Zurich 8092, Switzerland

**Keywords:** CMR_glc_, FDG, Fractional blood volume, Kinetic modeling, Reliability, Positron emission tomography, Infusion

## Abstract

**Background:**

Kinetic modeling of brain glucose metabolism in small rodents from positron emission tomography (PET) data using 2-deoxy-2-[^18^ F]fluoro-d-glucose (FDG) has been highly inconsistent, due to different modeling parameter settings and underestimation of the impact of methodological flaws in experimentation. This article aims to contribute toward improved experimental standards. As solutions for arterial input function (IF) acquisition of satisfactory quality are becoming available for small rodents, reliable two-tissue compartment modeling and the determination of transport and phosphorylation rate constants of FDG in rodent brain are within reach.

**Methods:**

Data from mouse brain FDG PET with IFs determined with a coincidence counter on an arterio-venous shunt were analyzed with the two-tissue compartment model. We assessed the influence of several factors on the modeling results: the value for the fractional blood volume in tissue, precision of timing and calibration, smoothing of data, correction for blood cell uptake of FDG, and protocol for FDG administration. Kinetic modeling with experimental and simulated data was performed under systematic variation of these parameters.

**Results:**

Blood volume fitting was unreliable and affected the estimation of rate constants. Even small sample timing errors of a few seconds lead to significant deviations of the fit parameters. Data smoothing did not increase model fit precision. Accurate correction for the kinetics of blood cell uptake of FDG rather than constant scaling of the blood time-activity curve is mandatory for kinetic modeling. FDG infusion over 4 to 5 min instead of bolus injection revealed well-defined experimental input functions and allowed for longer blood sampling intervals at similar fit precisions in simulations.

**Conclusions:**

FDG infusion over a few minutes instead of bolus injection allows for longer blood sampling intervals in kinetic modeling with the two-tissue compartment model at a similar precision of fit parameters. The fractional blood volume in the tissue of interest should be entered as a fixed value and kinetics of blood cell uptake of FDG should be included in the model. Data smoothing does not improve the results, and timing errors should be avoided by precise temporal matching of blood and tissue time-activity curves and by replacing manual with automated blood sampling.

## Background

The need for reliable quantification in positron emission tomography (PET) studies is self-evident: Data from different laboratories and from different subjects can be compared meaningfully only if the same numbers always mean the same thing. Clinical diagnostics and ethical issues like the use of the smallest possible number of laboratory animals depend on this. It is undesirable to acquire control group data every time a new condition or disease model is investigated. We have commented on the impact of inherent variability and experimental parameters on the reliability of small animal PET data in general [1], here we discuss more specific issues. The most commonly used tracer in PET is 2-deoxy-2-[^18^ F]fluoro-d-glucose (FDG). The simplest way to compare FDG datasets is by pseudo-quantification with standard uptake values (SUV). It has been argued that this approach might be both unreliable and uninformative [2]. Kinetic modeling represents a more complicated but preferable way of quantification. It yields kinetic information on the mechanisms underlying the tissue uptake of FDG. For the quantitative evaluation of FDG uptake into the target tissue, kinetic modeling takes into account the plasma FDG activity-time profile rather than the applied FDG dose as used for SUV. Results are, therefore, exclusively dependent on the uptake kinetics in the studied tissue and are not affected by inter- and intra-individual differences in systemic FDG disposition. The standard model of brain FDG uptake is the two-tissue compartment model, initially introduced as the autoradiographic method [3]. It requires a time-activity curve (TAC) from the tissue of interest and the arterial plasma time-activity curve, the so-called input function (IF), to derive single rate constants describing reversible transport across the blood–brain barrier (*K*_1_ and *k*_2_) and phosphorylation of FDG (*k*_3_) as well as hydrolysis of FDG-6-phosphate back to FDG (*k*_4_) [4]. The uptake rate constant of FDG (*K*_FDG_) can subsequently be calculated from the rate constants *K*_1_ to *k*_3_, as shown in Equation 1:

(1) $$K_{\mathrm{FDG}}=\frac{K_{1}\times k_{3}}{k_{2}+k_{3}}$$

The cerebral glucose metabolic rate (CMR_glc_) is estimated by dividing *K*_FDG_ by the lumped constant (LC), which corrects for the differences in the kinetics of FDG and glucose regarding their transport and phosphorylation, and multiplication by the arterial plasma glucose concentration (*G*_p_), as shown in Equation 2 [5]:

(2) $$\mathrm{CM}R_{\mathrm{glc}}=K_{\mathrm{FDG}}\times \frac{1}{\mathrm{LC}}\times G_{p}$$

Uncertainty about the absolute values of the individual rate constants from non-linear regression analysis with the two-tissue compartment model has relatively little effect on the calculated hybrid rate constant *K*_FDG_ and finally CMR_glc_[6]. To accurately determine the single rate constants, the following considerations need to be taken into account. (1) Both IF and TAC need to be recorded with short time intervals to cover rapid changes in activity in blood and tissue of interest. In particular, the IF after a bolus intravenous injection is characterized by rapid changes at the start of the experiment. High-frequency sampling of the IF has been challenging in rodents until recently but is now becoming state of the art [7,8]. However, non-homogenous FDG distribution in the blood pool during the first 30 s after a rapid injection can still lead to erroneous results as blood radioactivity is, in general, not determined directly in the tissue of interest [9]. Aside from a high sampling frequency, the exact temporal match of IF and TAC starting times is indispensable. (2) If radioactivity is measured in whole blood, the IF needs to be corrected for the hematocrit and the uptake kinetics of FDG into red blood cells [10]. (3) Measured tissue radioactivity requires the subtraction of the radioactivity in the blood vessels of the respective tissue [11,12]. While blood radioactivity is experimentally accessible, the exact fractional volume of blood in the tissue of interest (*v*_b_) is generally unknown. We consider these points as the major reasons for the large variability in the data reported on FDG kinetic modeling in rodent brain. Table 1 shows some of the recently published results from FDG studies, including claims that CMR_glc_ under isoflurane is at levels indicative of isoelectricity, i.e., a state without electric neuronal signaling [13].

**Table 1:** Literature results of two-tissue compartmental FDG kinetic modeling in small rodents

**Reference**	***K***_**1**_	***k***_**2**_	***k***_**3**_	***k***_**4**_	***K***_**FDG **_**(mL/min/cm**^**3**^**)**	***v***_**b **_**(%)**	**CMR**_**glc **_**(μmol/min/100 g)**	**Anesthesia**	**LC**	**Sp**
Millet et al. [12]	0.14 ± 0.05	0.19 ± 0.25	0.07 ± 0.05	0.005 ± 0.004	0.044 ± 0.013	-^a^	90.3 ± 27.6	Urethane	0.6	R
Wu et al. [14]	0.10 ± 0.03	0.21 ± 0.10	0.05 ± 0.02	0.015 ± 0.006	0.019 ± 0.005	0	21.5 ± 4.3	Isoflurane	0.625	M
Yu et al. [15]	0.22 ± 0.05	0.48 ± 0.09	0.06 ± 0.02	0.025 ± 0.010	0.024 ± 0.007	0	40.6 ± 13.3	Isoflurane	0.6	M
Mizuma et al. [13]	0.20 ± 0.02	0.39 ± 0.05	0.14 ± 0.02	0.015 ± 0.002	0.053 ± 0.013	0	39 ± 3	Awake	0.625	M
Mizuma et al. [13]	0.156 ± 0.009	0.329 ± 0.005	0.032 ± 0.006	0.009 ± 0.004	0.014 ± 0.004	0	13 ± 4	Isoflurane	0.625	M
Alf et al. [7]	0.27 ± 0.09	0.57 ± 0.10	0.08 ± 0.02	0.018 ± 0.004	0.035 ± 0.013	5.5	61 ± 11	Isoflurane	0.6	M

Units for *K*_1_ to *k*_4_ as in Figure 3, *K*_FDG_ in [mL/min/cm^3^] and CMR_glc_ in [μmol/min/100 g]. ^a^*v*_b_ included in the model. Sp, species; M, mouse; R, rat.

We have recently introduced an FDG infusion protocol for kinetic modeling in mice [7]. The purpose of administering FDG over several minutes by a constant infusion rate rather than by a rapid bolus was to overcome the problem of inhomogeneous distribution in the blood pool after rapid injection and to allow for longer time intervals in the recording of radioactivity in blood and tissue of interest. In this recent study, we made an assumption for *v*_b_ based on computed tomography measurements [16]. Here, we address the question whether *v*_b_ can be reliably fitted from the IF, TAC, and blood time-activity curve in mouse brain FDG kinetic modeling under the applied experimental conditions. In parallel, we investigate the influence of data smoothing and time delays between IF and TAC. In a next step, we assess the influence of different IF corrections for FDG uptake into blood cells. Finally, we evaluate by simulations whether our infusion protocol indeed tolerates lower sampling frequency of blood than bolus injection. This could be of advantage for manual blood sampling or the generation of image-derived IFs, in particular in longitudinal studies where shunt surgery for high-frequency blood sampling is not feasible. Based on our findings, we suggest some guidelines for mouse brain FDG kinetic modeling.

## Methods

### Data acquisition and kinetic modeling

We used experimental TACs and blood-activity curves from a previous study [7] with C57BL/6 mice (*n* = 5) with normal glycemia (plasma glucose 11.9 ± 4.0 mmol/L (6.7 to 16.9 mmol/L)) for our analysis. In brief, animals were under isoflurane (1.5% to 2%) anesthesia, and body temperature and respiratory frequency were controlled at 36°C to 37°C and approximately 90 cycles/min, respectively. FDG (10 to 14 MBq) was administered intravenously as a constant infusion over 4.0 to 5.3 min. Blood radioactivity was recorded with a coincidence counter (Twilite, Swisstrace GmbH, Zurich, Switzerland) on a shunt volume of approximately 60 μL with 1-s temporal resolution. List mode data were acquired for 45 min on a GE Healthcare/Sedecal (Madrid, Spain) eXplore VISTA PET/CT scanner in parallel.

Calibration of the coincidence counter with respect to the PET scanner was performed daily. A syringe containing approximately 1 MBq/cm^3^ FDG solution was attached to a catheter as used for the shunt [7], and FDG solution was flushed through the catheter which was guided through the coincidence counter. The syringe and catheter were measured simultaneously with scanner and coincidence counter, respectively. FDG radioactivity (Bq/cm^3^) was calculated from the images of the calibrated scanner and divided by the coincidence counts per cubic centimeter from the blood counter. This ratio was used to calculate blood radioactivity in the animal experiments.

**Figure 1:**
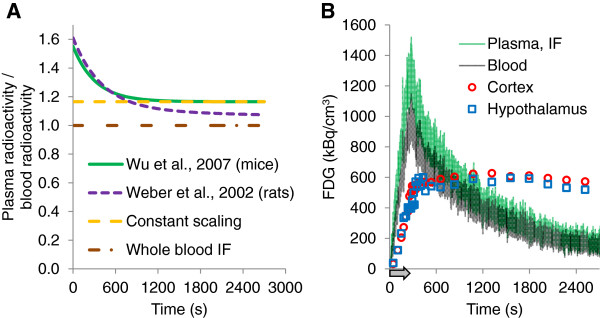
**Functions to generate IFs from blood radioactivity and data of a representative experiment. (A)** Exponential functions and scaling factor to calculate plasma form blood radioactivity. If not stated otherwise, the exponential function described in Equation 3 (Wu et al. [14], solid green line) was used in this study. For comparison, IFs were calculated with the bi-exponential function described for rats (Weber et al. [8]; dashed magenta line) and with a constant scaling factor corresponding to the equilibrium partition coefficient of plasma to blood (Wu et al. [14]; dashed orange line). Finally, whole blood was used as IF for comparison (constant factor 1; dashed-dotted brown line). **(B)** Representative data of an experiment (scan number V1131). Blood time-activity curve (grey), IF as calculated with the function in green in **(A)** (Wu et al. [14]; Equation 3), TAC of the cortex (red circles), and the hypothalamus (blue squares). Arrow indicates infusion duration.

If not stated otherwise, plasma radioactivity, i.e., the IF was calculated from the blood radioactivity with Equation 3 correcting for blood cell uptake kinetics in mouse [14,15]. For comparison, IFs were in addition calculated with Equation 4, which was determined for blood cell uptake kinetics in rats [8]. Furthermore, we calculated IFs from the experimental blood data by multiplication with the constant factor 1.165, which is the equilibrium partition coefficient determined by Wu et al. [14] (Equation 3). Finally, we also used the whole blood radioactivity as IF. The four functions of plasma to blood radioactivity (*A*_p_/*A*_b_) are plotted against time in Figure 1A.

(3) $$\frac{A_{p}}{A_{b}}=0.386\times e^{-0.191\times t\left(\min\right)}+1.165$$

(4) $$\begin{array}{ll} \frac{A_{p}}{A_{b}}= & 0.51\times e^{-0.1447\times t\left(\min\right)}+0.3\times e^{-0.00206\times t\left(\min\right)}\\ & +0.8 \end{array}$$

Image data were reconstructed into 33 to 39 time frames with the shortest frames (10 s) around the infusion stop and longer frames toward the end of the scan (maximal length 240 s) and analyzed with PMOD v3.4 (PMOD Technologies Inc., Zurich, Switzerland). TACs were derived from the cortex and hypothalamus, respectively, with anatomic templates of PMOD covering the entire structures. Figure 1B shows experimental TACs and IF of one representative experiment.

Two-tissue compartment kinetic modeling was performed with PMOD. A Marquardt-Levenberg algorithm was used for fitting until convergence to unique solutions. The LC for CMR_glc_ calculation was 0.6 and *v*_b_ 5.5% if not stated otherwise [7,16]. *χ*^2^ was used as indicator for goodness of fit according to Equation 5:

(5) $$\chi^{2}=\overset{n}{\underset{i=1}{\sum }}\left(\frac{{\left(O_{i}-E_{i}\right)}^{2}}{E_{i}}\right),$$

where *n* is the total number of observations (*O*_i_) and *E*_i_ is the expected value for *O*_i_ as calculated with the fit function.

### Influence of choice of fractional blood volume on fit parameters

The effect of different assumptions for *v*_b_ on the fitted model parameters and *χ*^2^ for the cortex and hypothalamus was tested by systematically varying *v*_b_ between 0 and 0.2 (i.e., 0% and 20% blood in tissue, in steps of 0.5%). Alternatively, *v*_b_ was included as a variable parameter in the fit. In order to reduce the impact of noise in the experimental data on *χ*^2^, smoothed TACs and IFs were generated from the whole experimental datasets with robust locally weighted regression (LOWESS) smoothing as implemented in MATLAB. Furthermore, to assess the effects of limited degrees of freedom, every second data point was deleted from the experimental TACs. Modeling was then performed as described above with fixed or variable *v*_b_ for each of the five scans with the following five combinations: unmodified experimental IF with unmodified TAC or with TAC after deletion of every second data point or with smoothed TAC, as well as smoothed IF with unmodified TAC or with smoothed TAC. To visualize the effect of the chosen *v*_b_ on CMR_glc_, CMR_glc_ values were normalized to the averaged CMR_glc_ over all *v*_b_ for each scan (CMR_glc_ at a particular *v*_b_/averaged value of all calculated CMR_glc_ of this scan) and plotted against *v*_b_. For the rate constants *K*_1_ to *k*_4_, average values from the five scans were plotted against *v*_b_.

### Influence of time delays between IF and TAC and of miscalibration between scanner and coincidence counter

To estimate the effect of time delays between TAC and IF, we shifted the experimental unmodified IF relative to the experimental unmodified TAC within a window of −20 to 30 s and plotted *χ*^2^ of the model fits (with constant *v*_b_ 0.055) against the timing error. To visualize the influence on CMR_glc_ and the single rate constants, the parameters were normalized to the respective value at zero time delay for each scan (e.g., CMR_glc_ (delay i)/CMR_glc_ (no delay)). To simulate a minor miscalibration by 5% between scanner and coincidence counter, the TAC was multiplied with 0.95 and 1.05, respectively. The resulting model fit parameters were compared to correctly time-matched and calibrated data fitting results.

### Fit of the IFs and simulations of IFs of a bolus and two infusion protocols

For further analysis and simulations, IFs were fit with the Solver add-in in Excel 2010 (Microsoft Office) with the tri-exponential functions shown in Equations 6 and 7 [17]

(6) $$\begin{array}{ll} C_{\inf}= & \left(A+B+Z\right)\\ & \times \left(f_{a}\left(1-e^{-\mathrm{\alpha t}}\right)+f_{\beta }\left(1-e^{-\mathrm{\beta t}}\right)+f_{z}\left(1-e^{-\zeta t}\right)\right) \end{array}$$

(7) $$\begin{array}{ll} C_{\mathrm{decr}}= & \left(A+B+Z\right)\left(f_{a},\left(1-e^{-\alpha t_{i}}\right),e^{-\alpha \left(t-t_{i}\right)}\right)\\ & +f_{b}\left(1-e^{-\beta t_{i}}\right)e^{-\beta \left(t-t_{i}\right)}\\ & \left(+f_{z}\left(1-e^{-\zeta t_{i}}\right)e^{-\zeta \left(t-t_{i}\right)}\right), \end{array}$$

where *C*_inf_ is the arterial plasma radioactivity during the infusion and *C*_decr_ the radioactivity after infusion stop (*t*_i_). The time point of infusion stop, *t*_i_, was determined from the curve maximum by visual inspection of the peak area of the IF. The sum of *A*, *B*, and *Z* corresponds to the extrapolated radioactivity in arterial plasma at steady state (infinite infusion duration). The fractional areas under the curves *f*_*a*_, *f*_*b*_, and *f*_*z*_ are defined by *A*, *B*, *Z* and *α*, *β*, *ζ*, as shown in Equations 8, 9, and 10:

(8) $$f_{a}=\frac{A}{\alpha \left(\frac{A}{\alpha }+\frac{B}{\beta }+\frac{Z}{\zeta }\right)}$$

(9) $$f_{b}=\frac{B}{\beta \left(\frac{A}{\alpha }+\frac{B}{\beta }+\frac{Z}{\zeta }\right)}$$

(10) $$f_{z}=\frac{Z}{\zeta \left(\frac{A}{\alpha }+\frac{B}{\beta }+\frac{Z}{\zeta }\right)}$$

Note that *A*, *B*, and *Z* are proportional to the infusion rate. *A*, *B*, *Z* and *α*, *β*, *ζ* were fit from the experimental IFs and kinetic analysis of the PET data was performed as described above with the fitted IF function. Fit FDG rate constants were compared to those with experimental IFs.

### Simulation of TACs and FDG kinetic modeling with different infusion protocols

IFs with bolus/infusion durations of 10 s (bolus), 300 s (similar to our experimental infusion protocol), and 900 s (for comparison) were simulated from the fit parameters *A*, *B*, *Z*, *α*, *β*, *ζ* with Equations 6 and 7 after adjusting *A*, *B*, and *Z* to the respective infusion rate (at equal FDG dose as in the experiment). The corresponding TACs were simulated with the PMOD software, applying the FDG two-tissue compartment model and *K*_1_, *k*_2_, *k*_3_, *k*_4_ from the fits with the experimental IFs and TACs with *v*_b_ 0.055. The number and minimal/maximal lengths of time frames for the simulated TACs were equal to the experimental data; however, shortest time frames were grouped around the corresponding injection/infusion stop. Blood radioactivities required for the correction with *v*_b_ were simulated from the generated IF according to Equation 3.

Once IFs and TACs were generated, Gaussian noise was added with the Excel function NORMINV to the simulated data. The standard deviation for noise generation of the IF consisted of a constant between 25 and 40 kBq/cm^3^ plus 4% to 6% of the simulated plasma concentration. For TAC simulations, a relative standard deviation was chosen for the Gaussian noise corresponding to the simulated TAC value multiplied with 0.8 and divided by the lengths of the time interval in seconds. These standard deviations yielded similar noise levels as observed for the experimental data. For each animal and infusion protocol, one IF and ten TACs were generated as described above, and kinetic modeling was performed with these simulated, noise-containing IFs and TACs as described above. Fit parameters were compared to the experimental values, and mean values and standard deviations of the fitted parameters were compared between the bolus and infusion protocols.

Finally, to investigate the influence of sampling frequency on the fit parameters and fitting precision (parameter standard deviations), IF sampling intervals were prolonged from the experimental 1 s to 30 s and 60 s, respectively, by deleting the data between these time points from both the experimental and above simulated noise-containing IFs. Kinetic modeling was performed with the identical simulated noise-containing TACs as used for the complete IF datasets.

### Statistical analysis

Data are presented as mean ± SD; error bars in figures represent SD and are further specified in the figure legends and text. Fitted parameters with the simulated IFs and TACs were compared by two-tailed homoscedastic *t* test. The effects of data smoothing and miscalibration were assessed with paired-sample *t* test, corrected for multiple comparisons (Bonferroni). Significant differences are indicated with an asterisk (*) for *P* < 0.05 and double asterisk (**) for *P* < 0.01.

## Results

### Fractional blood volume

**Figure 2:**
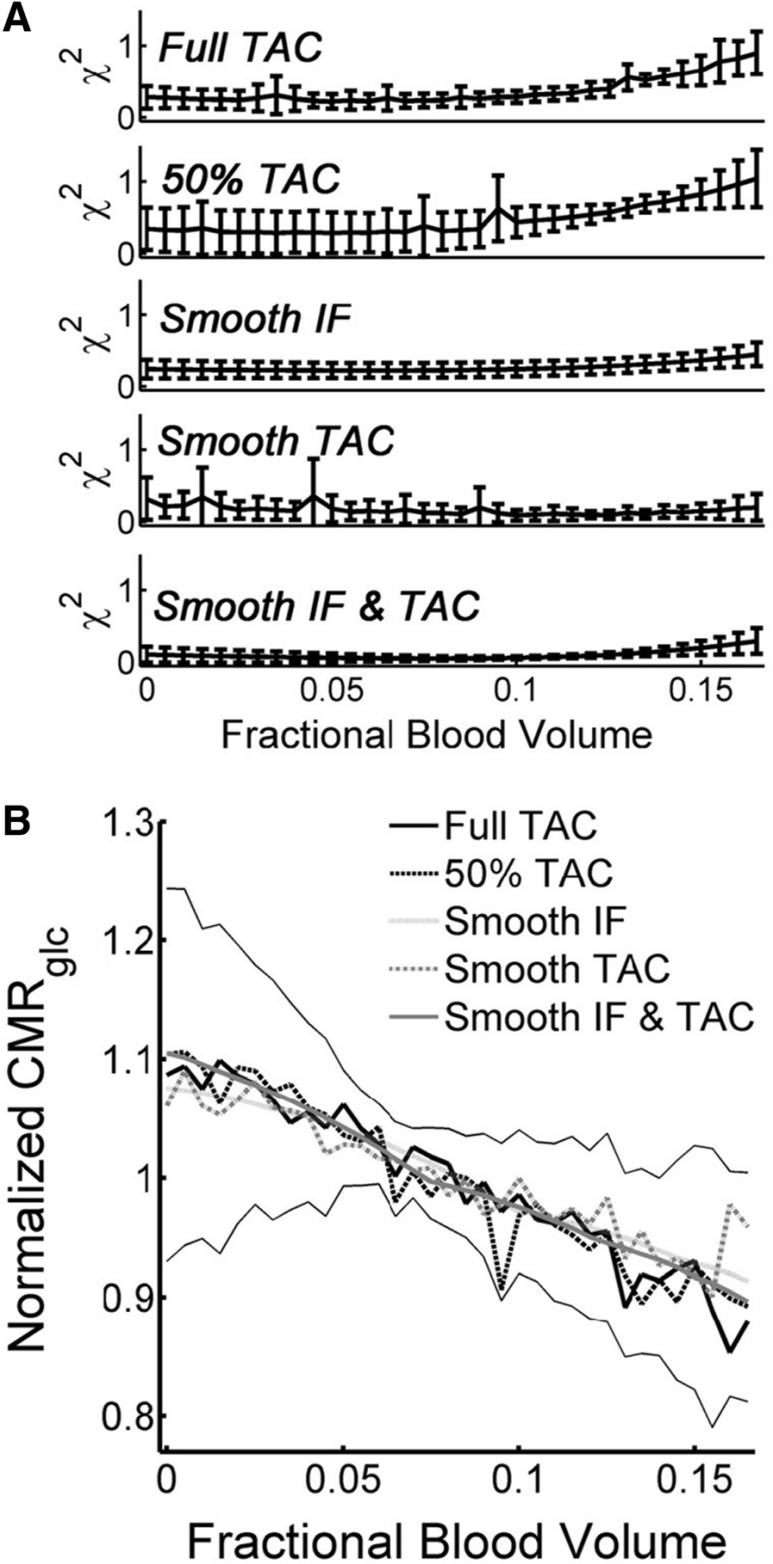
**Effect of fractional blood volume on modeling with data from the cortex. (A)** Effect on goodness of fit (*χ*^2^). Original data were more sensitive to unrealistically high *v*_b_ in the model. **(B)** Averaged CMR_glc_ was increased by about 10% when *v*_b_ was assumed to be zero. Thin lines are drawn one average standard deviation of the experimental data (*n* = 5 animals) above and below the average data curve.

To estimate *v*_b_ from the experimental FDG data, two-tissue compartment modeling was performed by varying *v*_b_ as a fixed value and by including *v*_b_ as a variable fit parameter, respectively. Analysis was performed with full experimental datasets of the five scans and with smoothed data and reduced TACs, respectively, as described under the ‘Methods’ section. Figure 2A shows the average *χ*^2^ of the modeling for the cortex with the five scans as a function of *v*_b_. Table 2 shows the *v*_b_ values resulting in lowest *χ*^2^ when varied between 0% and 20% as well as the fit values of *v*_b_ when included as fit parameter. In summary, data smoothing or reduction in TAC data had no significant influence on *v*_b_ yielding the lowest *χ*^2^ or on the fitted value for *v*_b_. Comparing the two brain regions, *v*_b_ was significantly higher in the hypothalamus than the cortex under three of the four fitting conditions (see Table 2). However, the respective *v*_b_ values in the hypothalamus were unrealistically high [16].

**Table 2:** **Fractional blood volume (*v***_***b***_**, in %) as estimated with the two-tissue compartment model**

		**Full TAC**	**50% TAC**	**Smooth IF**	**Smooth TAC**	**Smooth IF and TAC**
Cortex	Min. *χ*^2 a^	3.8 ± 4.0	4.6 ± 3.4	3.7 ± 3.9	7.8 ± 6.2	6.3 ± 1.9
	Model fit^b^	7.2 ± 4.0	9.7 ± 7.2	3.7 ± 3.8	15.1 ± 7.1	6.3 ± 1.8
Hypothalamus	Min. *χ*^2^	12.7 ± 2.7^c^	Not fitted	Not fitted	Not fitted	11.1 ± 5.0
	Model fit	11.7 ± 1.1^c^				11.5 ± 4.1^c^

^a^Min. *χ*^2^, *v*_*b*_ was varied manually to yield the best goodness of fit (minimal *χ*^2^). ^b^Model fit, *v*_*b*_ was included as model parameter to be fitted. ^c^Significant difference to respective value for the cortex but unrealistically high.

**Figure 3:**
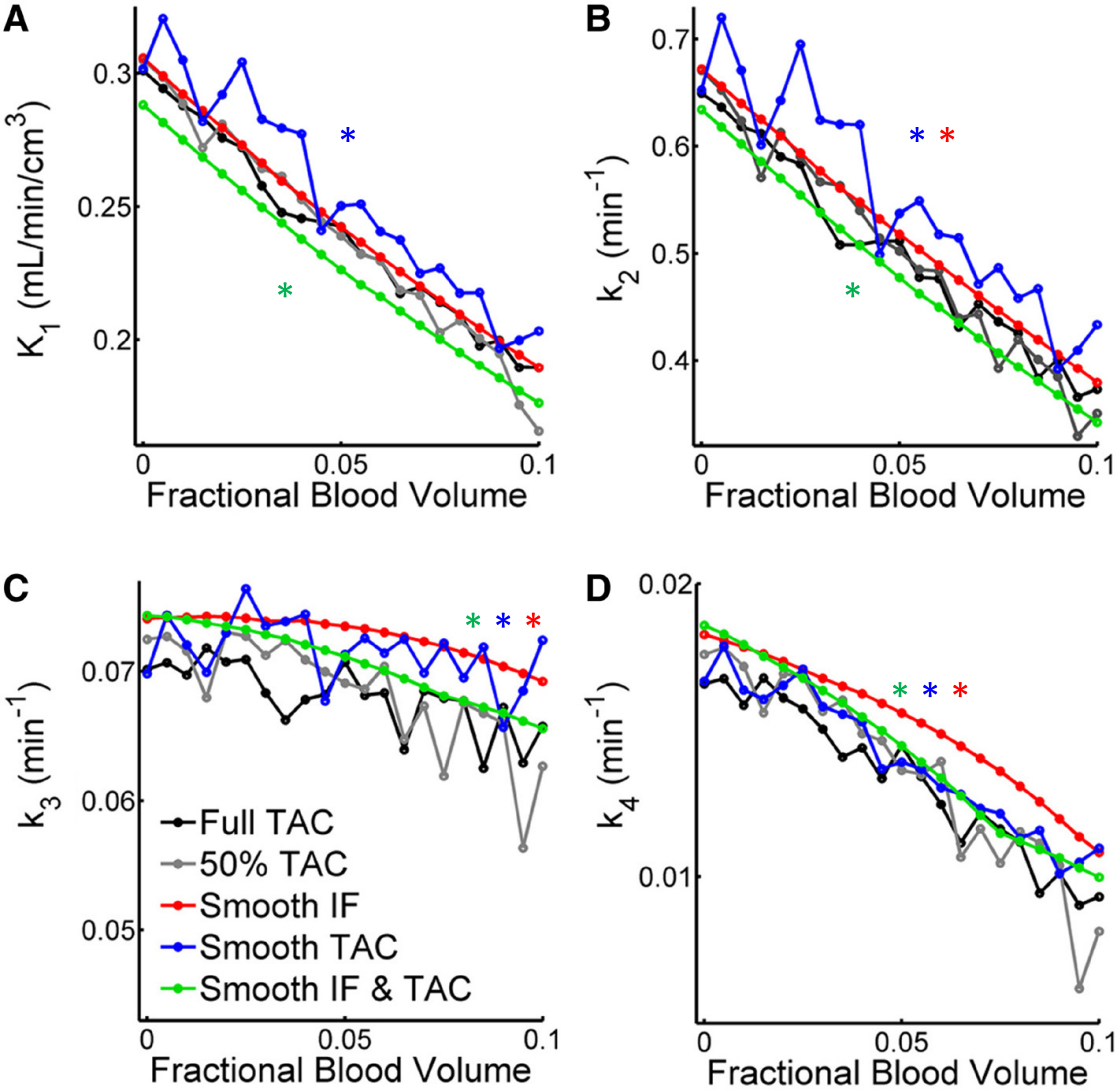
**Effect of fractional blood volume on the single rate constants.***K*_1_**(A)** and *k*_2_**(B)** showed an almost linear relationship in the cortex, while the pattern for *k*_3_**(C)** and *k*_4_**(D)** was more complex. Effects were independent of the brain region. Single asterisk (*) denotes the results from model fits using smoothed data deviated significantly (*P* < 0.05) from those achieved with the full TAC. Error bars are omitted for better readability.

As shown in Figure 2B, the average value of CMR_glc_ varied by ±10% when varying *v*_b_ between 0% and 15%. Individual values of CMR_glc_ deviated from the mean by >20% at very low or high values of *v*_b_ as indicated by the error bars in Figure 2B. Figure 3 shows the dependency of the single rate constants on the chosen value for *v*_b_. All single rate constants negatively correlated with *v*_b_. Changes of *v*_b_ by 0.5% resulted in differences of up to 15% in individual rate constants.

### Influence of data smoothing on the fitted rate constants

Figures 2B and 3 show the influence of data smoothing on CMR_glc_ and rate constants. Data smoothing did not affect CMR_glc_ significantly, but significant differences occurred between estimates of all single rate constants with the original TAC and their estimates achieved with any combination of the smoothed data vectors (*P* < 0.05) with only one exception (comparison *K*_1_ with smooth IF versus original data, see Figure 3A). In general, data smoothing leads to underestimation of *K*_1_ and *k*_2_ by 5% and 4%, respectively, when both TAC and IF were smoothed, and to overestimation by 5% to 15% when only TAC or IF were smoothed.

### Delay between IF and TAC, calibration errors

**Figure 4:**
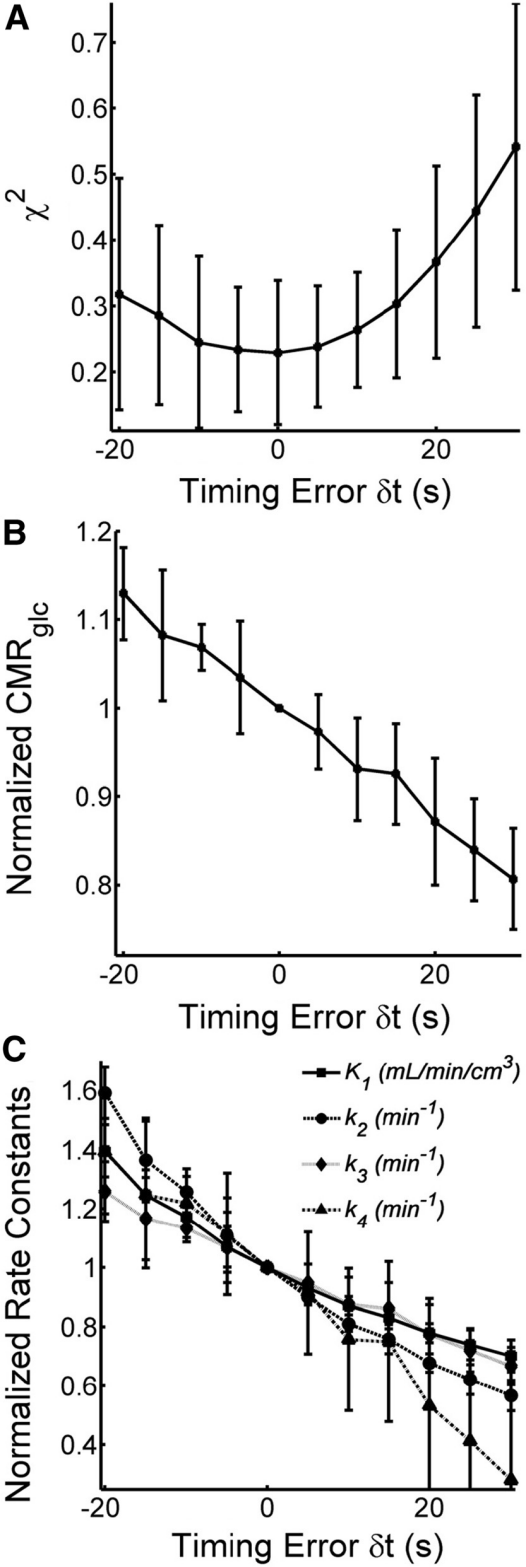
**Effect of blood sample timing errors. (A)** Goodness of fit was best at no delay between IF and TAC but significantly decreased within the investigated range of time delay. **(B)** CMR_glc_ was sensitive to timing errors. A delay of as little as 5 s in either direction resulted in significant changes. **(C)** Timing errors led to large deviations in all single rate constants. Average values with standard deviations from five animals each.

Figure 4 shows the influence of delays between TAC and IFs on the fit parameters. Goodness of fit was best at zero delay between TAC and IF. Timing errors affected CMR_glc_ and the single rate constants. As little as a 5-s delay in either direction resulted in significant (*P* < 0.05) over- or underestimation of CMR_glc_ (Figure 4B). The effect on CMR_glc_ was brought about by changes in all single rate constants. Figure 4C shows the single rate constants normalized by their respective value at zero timing error. Timing errors of 20 s resulted in parameter estimate errors of up to 60%.

Table 3 shows the consequences of calibration errors between scanner and coincidence counter. Miscalibration of 5% resulted in significantly wrong estimates of *K*_1_ (−7 ± 3% and +6 ± 4% for too low and too high scanner/coincidence counter calibration factors, respectively) and CMR_glc_ (−5 ± 2% and +6 ± 4%), without affecting the other rate constants. Goodness of fit was decreased but not significantly.

**Table 3:** Relative changes in model estimates due to calibration errors

**Calibration error scanner/counter**	***K***_**1**_	***k***_**2**_	***k***_**3**_	***k***_**4**_	**CMR**_**glc**_	***χ***^**2**^
−5%	−7 ± 3%*	−1 ± 6%	+1 ± 5%	+4 ± 8%	−5 ± 2%*	+ 9 ± 24%
+5%	+6 ± 4%*	+1 ± 6%	0 ± 6%	+4 ± 12%	+6 ± 4%*	+10 ± 28%

After correction for multiple comparisons, only the differences in *K*_1_ and CMR_glc_ were significant. *P < 0.05.

### Correction for FDG blood cell uptake

**Figure 5:**
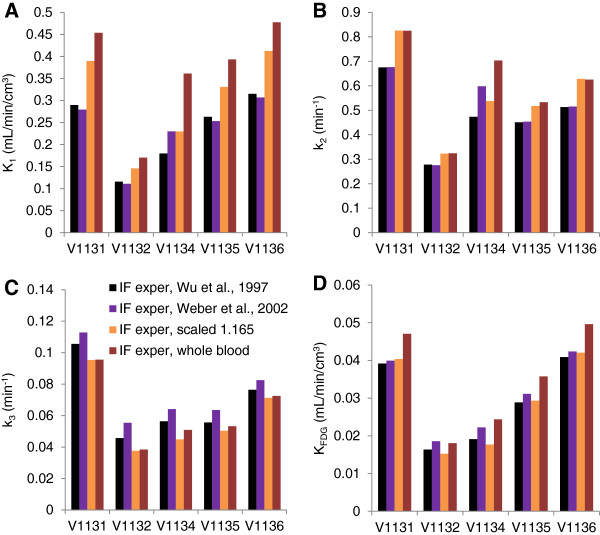
**Influence of the correction for blood cell uptake on FDG kinetic modeling in mouse brain cortex. (A)** K_1_, **(B)** k_2_, **(C)** k_3_, **(D)** K_FDG_. V1131 to V1136 are scan numbers. IFs were calculated according to Equation 3 (black bars; IF exper, Wu et al. [14]; for mice), Equation 4 (magenta; IF exper, Weber et al. [8]; for rats), with a constant scaling factor (light brown; IF exper, scaled 1.165) or blood radioactivity was used as IF (dark brown).

IFs were calculated from the experimental blood radioactivity data by four methods, two exponential correction functions published for mice (Equation 3) and rats (Equation 4), taking into account uptake kinetics into blood cells, and by scaling the blood activity-time curve by a constant plasma/whole blood partition coefficient or using the blood curve as the IF. The resulting rate constants of the five scans are shown in Figure 5. In general, the two exponential functions for mice and rats revealed similar rate constants, both for the cortex (Figure 5) and hypothalamus (not shown). Applying the whole blood radioactivity-time curve as the IF resulted in an overestimation of *K*_1_ in the cortex and hypothalamus between 46% and 106% as compared to the parameters calculated with the IF according to Equation 3. The rate constants *k*_2_ in the cortex and hypothalamus were between 9% and 51% higher with the whole blood IF. All *k*_3_ values were lower with the whole blood IF; they resulted to between 68% and 95% of the reference values. Finally, *K*_FDG_ in the cortex and hypothalamus calculated with the whole blood IF was between 96% and 129% of the reference data. Constant scaling with the equilibrium partition coefficient increased *K*_1_ but to a lower extent than applying the whole blood curve. The *k*_3_ values were similar with the whole blood IF or the scaled IF in both the cortex and hypothalamus. Finally, scaling resulted in similar CMR_glc_ values as the correction according to Equation 3; values were within 84% and 103% of the reference values in the cortex and hypothalamus.

### Non-linear regression analysis of experimental IF and simulations of IF and TAC

**Figure 6:**
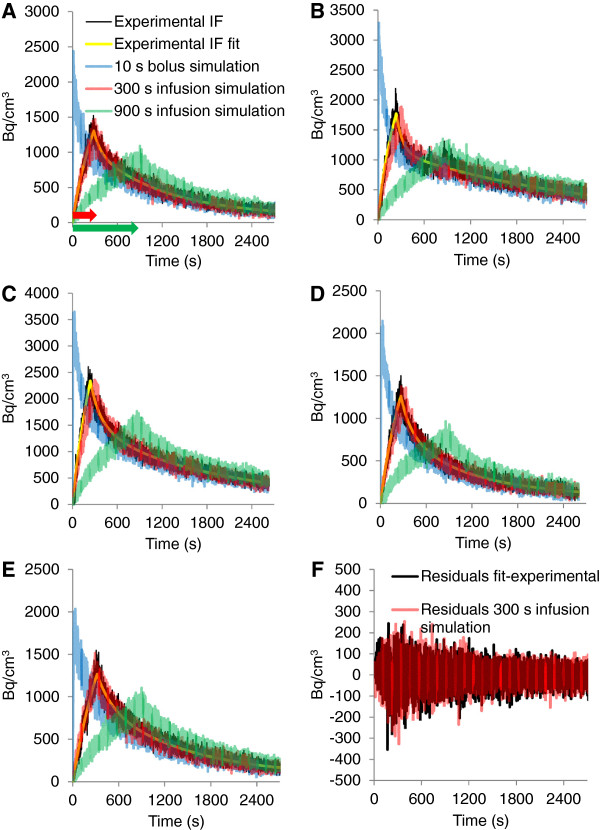
**Experimental, fit, and simulated IFs. (A to E)** Experimental IF (black), tri-exponential fit according to Equations 6 and 7 (yellow), simulation 10-s bolus injection (blue), simulation 300-s constant infusion (red), simulation 900-s constant infusion (green) for scans V1131 **(A)**, V1132 **(B)**, V1134 **(C)**, V1135 **(D)**, and V1136 **(E)**. **(F)** Residuals between experimental IF and fit function in black and between simulated IF (300-s infusion) with and without noise in red (scan V1131). Arrows in **(A)** indicate the duration of the infusions.

To simulate IFs for bolus and infusion protocols, the experimental IFs were fitted with Equations 6 and 7. Figure 6 shows the agreement between fit and experimental IFs. Based on the fit parameters, we simulated IFs for bolus FDG injection (injection over 10 s) and constant infusions over 300 s (similar to the experimental infusion protocols) and 900 s. In a next step, we added Gaussian noise to the simulated IFs. The resulting IFs are shown in Figure 6. To study the influence of sampling frequency on FDG kinetic parameters, we reduced the IF data to data points in 30- and 60-s intervals, respectively. The same was done with the experimental IFs.

TACs for the cortex were generated with PMOD from the simulated IFs (before noise was added) and the experimental rate constants. From each generated TAC, ten variations were calculated by the addition of random Gaussian noise as described under the ‘Methods’ section.

### FDG kinetic modeling with simulated bolus and experimental and simulated infusion protocols

**Figure 7:**
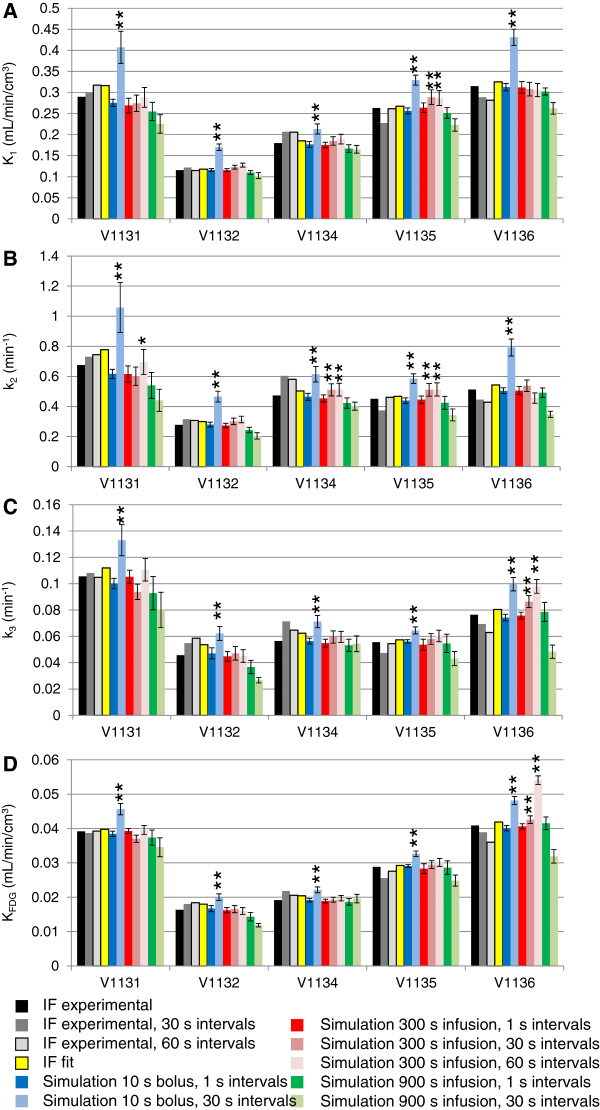
**Fit parameters generated with experimental and simulated IFs and TACs. (A)***K*_1_, **(B)***k*_2_, **(C)***k*_3_, **(D)***K*_FDG_. IF exper as in Figure 5 (black bars). Experimental IFs were reduced to data points every 30 s (dark grey) and 60 s (light grey), respectively, and experimental TACs were fitted with the reduced IFs. Data were in addition calculated with the fitted IF and experimental TACs (yellow). Bolus injection over 10 s simulated with 1-s (dark blue) and 30-s (light blue) sampling intervals. Simulated infusion protocols over 300 s with 1-s (red), 30-s (medium red), and 60-s (light red) sampling intervals. Simulated infusion over 900 s with 1-s (dark green) and 30-s (light green) sampling intervals. Simulated data are averages of fits with ten simulated TACs each, error bars indicate the standard deviations of the ten fits. All simulated IFs and TACs contained Gaussian noise, except of the fit IF (yellow). Single and double asterisk (* and **) denote some significant differences to the respective rate constants of the 300-s infusion protocol, 1-s sampling interval at P < 0.05 and *P* < 0.01, respectively. Note that most rate constants of the 900-s infusion protocol, 30-s sampling interval, were significantly (*P* < 0.01) lower than the corresponding rate constants at 300-s infusion, 1-s sampling. Not all single asterisk (*) and double asterisk (**) are indicated for clarity.

As shown in Figure 7, FDG rate constants of simulated bolus and 300-s infusion protocol, both at 1-s blood sampling intervals, were similar to the rate constants determined with experimental IFs (1-s blood sampling interval). We first investigated whether kinetic modeling with the experimental IF including only data points at every 30 and 60 s, respectively, resulted in similar results as with the complete IF with sampling frequency of 1 s. As shown in Figure 7 for the cortex, *K*_1_, *k*_2_, *k*_3_, and *K*_FDG_ were within 82% and 128% of the reference values in the cortex and hypothalamus (not shown). We next investigated the influence of blood sampling frequency with the simulated data for the 10-s bolus administration. As expected, the 30-s sampling interval resulted in an underestimation of the peak radioactivity at the end of the injection (not shown). The effect on the rate constants was striking as shown in Figure 7. All rate constants with 30-s sampling intervals for the bolus administration were significantly higher than the rate constants simulated with the 10-s bolus protocol and 1-s sampling intervals (all with *P* < 0.001 except of one *k*_3_ value). In contrast, sampling frequency had only a minor effect on the fit parameters in the case of the 300-s infusion simulations, despite some significant differences between the fits with different sampling intervals. Standard deviations of the fit parameters with the 10-s bolus and 300-s infusion protocols at 1-s sampling interval were not significantly different. Fitting precision was thus not significantly better with one or the other protocol.

**Figure 8:**
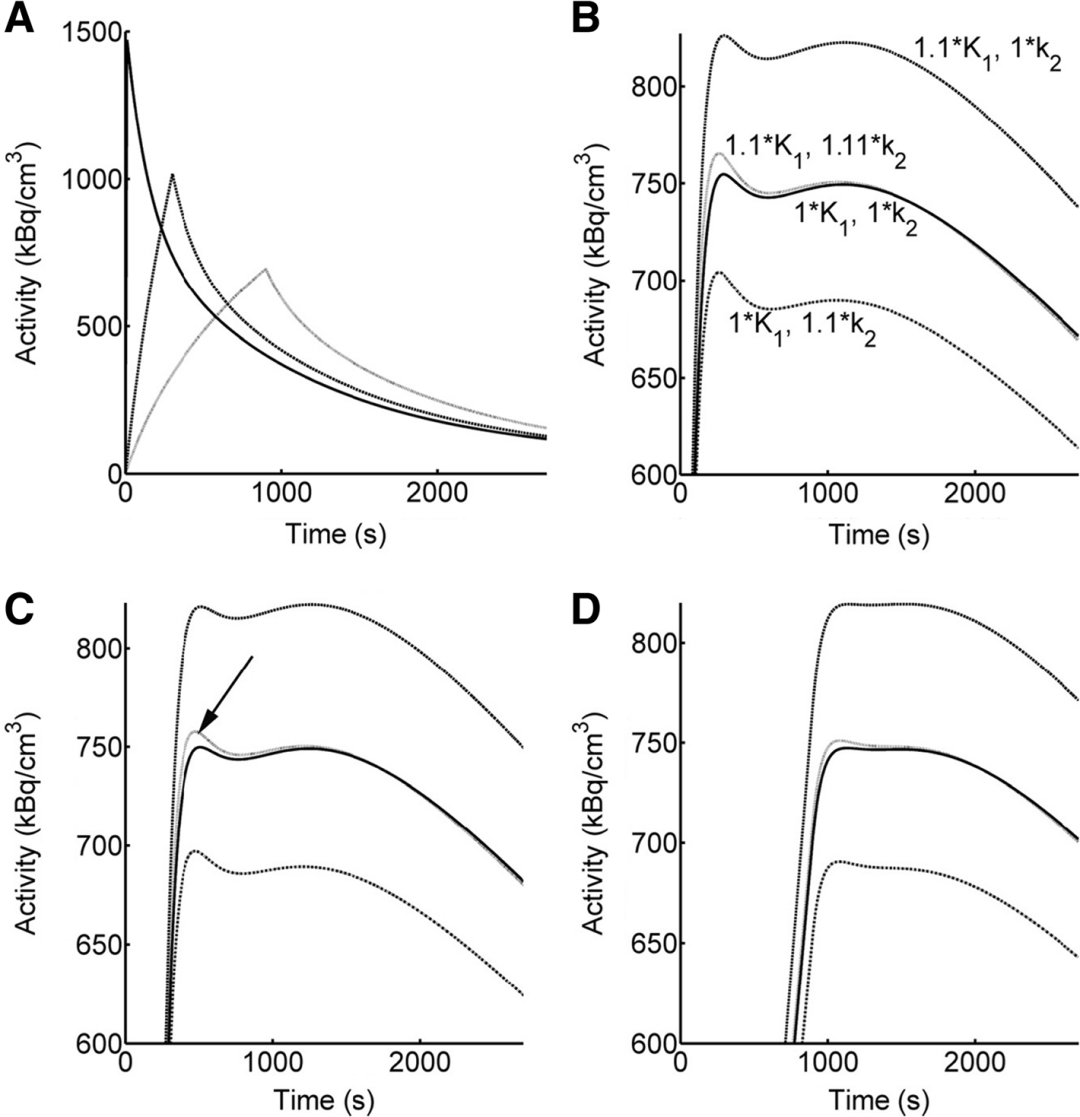
**Loss of information by prolonging the infusion duration. (A)** Simulated IFs, bolus 10 s (dark line) and infusions 300 (dotted line) and 900 s (grey line). **(B)** Zoom into TAC generated with a typical set of rate constants *K*_1_ 0.328 mL/min/cm^3^_,_*k*_2_ 0.550 min^−1^, *k*_3_ 0.079 min^−1^, *k*_4_ 0 (dark line). An additional TAC was generated by increasing *K*_1_ by 10% (1.1 × *K*_1_). At the same time, *k*_2_ was also increased to match the original TAC as close as possible. The respective factor for *k*_2_ was 1.11 (1.11 × *k*_2_). TACs with either increased *K*_1_ or *k*_2_ are shown in addition. The newly generated TAC with 1.1 × *K*_1_ and 1.11 × *k*_2_ deviates from the original TAC around the infusion stop, i.e., around the peak of the IF. This difference is indispensable to distinguish between the effects of *K*_1_ and *k*_2_ on the TAC and thus for kinetic modeling. **(C)** TACs generated with the same rate constants as in **(B)** for the 300-s infusion and **(D)** for the 900-s infusion. The difference between the two TACs (indicated by an arrow in C) reduces as infusion duration increases, explaining the improper fit parameters with the simulated 900-s infusion protocol at longer sampling intervals. Note that **(B)** to **(D)** zoom into the TAC region of interest and the activity scale, therefore starts at 600 Bq/cm^3^.

We extended the infusion duration to 900 s in the simulations to investigate whether rate constants can still be determined at longer infusion durations or whether precision decreases with infusion duration. Overall, rate constants were similar to the experimental values when sampling at 1-s intervals (in average *ca*. 10% lower). However, rate constants were on average up to 26% lower (*k*_2_ and *k*_3_) than the experimental parameters (significant for most constants but not indicated as such in the figure for clarity). Figure 8 gives an explanation why the infusion duration cannot be prolonged without losing information for the model fitting. We simulated scans with IFs for bolus (10 s) and infusions of 300- and 900-s duration as above (Figure 8A). Figure 8B,C,D zooms into the simulated TAC regions that are most important for fitting *K*_1_ and *k*_2_. We simulated TACs with typical rate constants from the experiments (dark lines) and then increased *K*_1_ by 10% (1.1 × *K*_1_) and increased *k*_2_ at the same time to match the newly generated TAC to the original TAC as close as possible. The respective factor for *k*_2_ was 1.11. The remaining difference between the two TACs is most prominent around the infusion stop (arrow in Figure 8C). This remaining difference is indispensable to distinguish the effects of *K*_1_ and *k*_2_ on the TAC. As shown in the figure, the difference between the two TACs dissipates as the infusion duration increases. This illustrates how information is lost with prolongation of infusion and the influences of *K*_1_ and *k*_2_ start to be indistinguishable.

## Discussion

Glucose uptake and metabolism and, therefore, FDG kinetics depend on many physiological factors. Minor deviations in physiological conditions can result in significant differences between the results from FDG PET studies [1,18,19]. However, without kinetic modeling and resolution of the single process rate constants of FDG and glucose in the region of interest, it is impossible to conclude whether such differences result from divergences in systemic FDG disposition, inconsistent data analysis or, indeed, differences in glucose transport and phosphorylation in the region of interest. In this work, we applied two-tissue compartment kinetic modeling to exclude the influence of systemic FDG disposition on the results. We focused on the application protocol and parameters that influence data analysis in kinetic modeling once the experimental part is completed. We show that several parameters and conditions which are often not paid much attention for can strikingly affect the modeling results and may lead to erroneous conclusions when comparing experimental results.

We showed that omission of *v*_b_ in the model equation and even minor flaws in experimental meticulousness can result in substantial distortions of FDG PET kinetic modeling results. Transport parameters were most sensitive to such methodological flaws, but *k*_3_ and *k*_4_ were also affected, and even the allegedly robust CMR_glc_ was subject to substantial changes. The sensitivity of the transport parameters to *v*_b_ and timing errors is a consequence of the shape of the IF: Correct estimation of *K*_1_ and *k*_2_ relies on the early time points of the IF and TAC, where equilibration between blood plasma and the free tissue pool occurs.

Our calculations suggest that it is desirable to fix *v*_b_ for model fitting rather than including it as a fit parameter, even with more than sufficient data points in IF and TAC, as its inclusion in the model did not lead to increased model precision or even an accurate fit of *v*_b_ itself. The goodness of fit carried little information about *v*_b_ and the fit estimates obtained for *v*_b_ are unlikely to reflect the true physiological situation. Even the inclusion of larger vessel structures such as the circle of Willis in the hypothalamic region of interest would not lead to a *v*_b_ of approximately 12% as suggested by modeling. We, therefore, recommend the use of literature values, e.g., from Chugh et al. [16] for *v*_b_ in the brain region under investigation. The situation may be different for bolus administration of FDG. At the very start of the scan, when the IF reaches its peak, tissue radioactivity is still low and *v*_b_ may be better accessible than with our infusion protocol.

Data smoothing did not result in greater precision of parameter estimates but affected estimates of single rate constants significantly. Smoothing the TAC removed at least some information related to *v*_b_: The best goodness of fit was shifted away from that of the original data fit along the *v*_b_ axis. Interpolation without curve smoothing may be the best option, if additional degrees of freedom are required for robust modeling.

Delay between starting times of IF and TAC and timing errors for early blood samples are likely to be among the major causes of variation in previous reports. Without automated sampling tools such as beta probes [20] or coincidence counters operating on a shunt volume [7,8], it is virtually impossible to get correctly timed samples because of catheter dead volume and the time needed for transfer between animal and measurement device or for blood plasma separation. We, therefore, recommend the substitution of manual sampling with automated, high temporal resolution sampling and to pay particular attention to synchronization of starting time of IF and TAC.

As expected from the impulse response function, systematic errors resulting in the multiplication of the input function by a constant factor, such as calibration errors, can only be compensated by *K*_1_. Rate constants *k*_2_ to *k*_4_ are in the exponents of the response function, defining the shape of the TAC [3], which is not affected by this kind of error.

The method how the IF is derived from the experimental blood data affects the single rate constants and CMR_glc_. Based on our results, kinetic modeling with whole blood IF or a constant scaling factor is not recommended. Our comparison of two similar correction functions suggests that minor inter- and intra-individual differences in blood cell uptake of FDG may affect the results of kinetic modeling. This should be taken into account, and individual kinetics of blood cell uptake should be determined when comparing FDG kinetics of a heterogeneous group of animals.

Our well-defined experimental IFs support the notion that infusion instead of bolus injection avoids unpredictable blood activities at the early time points due to non-instant distribution of the FDG in the central, that is, the measurement and input compartment [9]. We conclude from our simulations that loss of information for modeling is negligible at moderate infusion duration of 5 min as compared to bolus administration. Based on our simulation, a 5-min infusion protocol allows for longer intervals between blood samples than a bolus injection. The infusion protocol could thus allow kinetic modeling under conditions where a shunt surgery is not advisable and alternative less invasive blood sampling techniques are required, e.g., in longitudinal studies. We have successfully applied the infusion protocol for FDG kinetic modeling with image-derived IFs [7]. Here, we demonstrate in addition that infusion duration cannot be prolonged further without loss in information for kinetic modeling.

We may have missed a fast initial distribution phase in the simulated IFs of the bolus injection. The simulated IF of bolus V1132 resembled closest the general shape of an FDG bolus IF [15]. Results of this dataset were in agreement with those of the other simulations. For this reason and because an additional peak of short duration would add to the overestimation of *K*_1_ and possibly other rate constants if sampling intervals are not shortened, we conclude that our interpretation of the simulated data with the 30-s sampling intervals are correct.

There are two other pertinent issues in the quantification of CMR_glc_ which are not addressed in this work. One of them is the LC, which corrects for differences in the kinetics of FDG compared to glucose [5]. The LC can be derived from kinetic modeling results [21], but it does not influence the rate constants of FDG determined in this work (i.e., *K*_1_ to *k*_4_ and *K*_FDG_). The data in Table 1 all use similar LCs and can, therefore, be directly compared to each other. The second issue is the inclusion of the parameter describing dephosphorylation, *k*_4_, in the model [4]. As the results for other parameters were not significantly affected by *k*_4_ (which was close to zero), we decided to include it in the model for the present study.

## Conclusions

To increase the reliability of FDG PET data modeling with the two-tissue compartment model, we recommend FDG infusion over about 5 min; to include an appropriate value for the fractional blood volume in the tissue of interest as well as to correct the IF for blood cell uptake kinetics. Data smoothing was demonstrated to be an inappropriate manipulation, prohibiting precise and accurate modeling. We also show that delays of a few seconds between the start and early sampling of IF and TAC can lead to substantial misestimates. We hope that our findings will contribute toward improved methodological standards in FDG kinetic modeling.

## Competing interests

The authors declare that they have no competing interests.

## Authors’ contributions

MFA performed the experiments, analyzed and interpreted the data, and conceived and drafted the manuscript. MIMK and RS were involved in the conception of the manuscript, data interpretation, and manuscript writing. SDK contributed to data analysis and interpretation, to the conception of the manuscript, and to its writing. All authors read and approved the final manuscript.
